# Case report on trial: Do you, Doctor, swear to tell the truth, the whole truth and nothing but the truth?

**DOI:** 10.1186/1752-1947-5-179

**Published:** 2011-05-13

**Authors:** Oded Yitschaky, Michael Yitschaky, Yehuda Zadik

**Affiliations:** 1 Departments of Orthodontics, Oral Medicine, Hebrew University-Hadassah School of Dental Medicine, P.O.Box 91120, Jerusalem, Israel; 2 Oral Medicine, Hebrew University-Hadassah School of Dental Medicine, P.O.Box 91120, Jerusalem, Israel; 3 Editorial Board, Journal of Medical Case Reports

## Abstract

We are in the era of "evidence based medicine" in which our knowledge is stratified from top to bottom in a hierarchy of evidence. Many in the medical and dental communities highly value randomized clinical trials as the gold standard of care and undervalue clinical reports. The aim of this editorial is to emphasize the benefits of case reports in dental and oral medicine, and encourage those of us who write and read them.

## Editorial

We live today in the era of "evidence based medicine", in which our knowledge is stratified from top to bottom in a hierarchy of evidence [1-3]. At the pinnacle of this hierarchy we find randomized clinical trials, systematic reviews and meta-analyses, which are supposed to be the cream and cherry of medical-scientific-modern knowledge. Far below at the bottom of the pyramid we find case reports, which are often barely regarded as evidence. The aim of this editorial, however, is to emphasize the benefits of case reports, and encourage those of us who write and read them.

Randomized clinical trials are expensive, take years to conduct, and may encounter ethical problems, such as knowingly withholding treatment from a sample of patients. It is true that randomized clinical trials can give us a statistical answer for very narrow clinical questions, which can guide us when treating the "average patient" (i.e. the patient who comes with only the specific medical problem). As clinicians we know that most of our questions are not clearly answered in a randomized clinical trial, and many clinical questions cannot be answered. Clinical trials may be hindered by ethical restraints [4], financial limitations, and statistical factors; in some rare medical syndromes [5,6], it may be impossible to collect adequate samples to conduct a randomized clinical trial. With this in mind, it is interesting to read the Collins and Pinch report about how azidothymidine was approved as effective treatment for Acquired Immune Deficiency Syndrome (AIDS), even though the randomized clinical trials were stopped by the social and political pressure of AIDS activists [7]. One of the methodological requirements of a randomized clinical trial is double blindness, which means that both the patient and the physician do not know if that patient is receiving the treatment being evaluated or a placebo substitute. In 1997, Shapiro and Shapiro asked the question "How blind is blind?", and claimed that in randomized clinical trials the doctor can differentiate between true drug and placebo in about 80% of the cases, and the patient can differentiate in about 70% of the cases [8].

The case report can be published quickly [9], and be authored by busy clinicians who see patients on a daily basis and do not have the time or money to conduct large scale research. In our humble opinion, the experience of these clinicians is valuable infrastructure on which medical knowledge can be built. Additionally, randomized clinical trials can only inspect one variable or very few variables at the most and rarely reflect the full picture of a complicated medical situation. The case report can detail many different aspects of the patient's medical situation, specifically including patient history, physical examination, psychosocial aspects, follow up, etc.

With regards to the question: "can the medical community learn from one case?", our answer is that the medical community must learn from any case, especially those that are particularly unusual. These cases will give us, as clinicians, better insight into the unusual riddles which we usually encounter in our everyday practice. Let us leave the "hierarchy" for the bureaucratic institutions and the "evidence" for the court-rooms, and use our imagination and intuition - which are well documented in interesting case reports - to help us better treat our patients.

## Competing interests

The authors declare that they have no competing interests.

## Authors' contributions

OY, MY and YZ conducted this Editorial together.
